# Dr. S. P. Hullihen’s New Method of Filling Teeth over Exposed Nerves

**Published:** 1852-10

**Authors:** E. B. Gardette


### *Dr. S. P. Hullihen's New Method of Filling Teeth over exposed Nerves. Communicated by* E. B. Gardette, M. D.

Among the serious difficulties that have belonged to surgical operations for the preservation of the teeth, is that of successfully filling cavities formed by disease, after they have penetrated to the internal or natural cavity, and exposed the nerve.

Various expedients have been resorted to at different periods in the history and progress of the profession, to overcome this difficulty—such as, capping the nerve with a metallic plate, destroying it by means of the actual cautery, or by the application of caustics, and the entire extirpation of the nerve to the extremity of the fang. Each and all of these plans have had their trial and preference with more or less success in different hands, and oftener in very *indifferent* ones; but no candid and intelligent operator, I think, has regarded any of these modes of proceeding *unobjectionable*, whether viewed in reference to the results and advantages of the operation to the patient, or the surgical principles involved, and which the true surgeon should never lose sight of in his efforts to overcome disease.

I therefore propose to describe briefly the operation of Dr. Hullihen, of Wheeling, Va., or, in other words, his mode of treating a tooth, where the decay has reached the nerve—a mode which has proved most singularly successful in his own able hands for a number of years past, and one that, having myself fully tested for more than a year, I now adopt in my own practice in all cases where the condition of a tooth renders it proper.

After the removal of the diseased parts from a tooth has been accomplished, making it apparent that the nerve is exposed, the operator will proceed to perforate the fang into its natural or nerve-cavity, through the gum and outer plate of the alveolar process; the perforation to commence about a line or a line and a half from the free edge of the gum, and to be in size that of a small knitting-needle. The opening should be made with a view of doing no other violence to the nerve than that of opening its blood-vessels, which may be at once known by the flow of arterial blood. The cavity in the tooth, formed by decay, may then be immediately filled, without the least fear of pain or ill consequences of any kind arising from the pressure of the plug upon the nerve.

The physiological change effected by this operation upon the nerve, may not as yet, perhaps, be satisfactorily explained. That the operation prevents subsequent pain and inflammation in the nerve, and therefore preserves the vitality and color of the tooth, under circumstances which no other known course of treatment can effect, all experience thus far proves perfectly conclusive; and upon this simple fact the merits of the operation are now urged, and laid before the medical profession.

This original mode of treating an exposed nerve, has, with the consent of Dr. Hullihen, been communicated to the "American Society of Dental Surgeons," at their recent annual meeting at Newport, R. I., by Dr. C. O. Cone, Dentist, of Baltimore; but never having myself been a member of that Society, I trust it is in no self-conceit of the comparative importance of my professional opinions that I now perform this agreeable act of personal friendship through a medical journal.

With the characteristic modesty of genius, Dr. Hullihen would, I well know, forbid any attempt to express my sense of the benefits he has conferred upon the profession and those who need its relief; and yet as one of those who appreciate the difficulties belonging to the class of operations under consideration, I must regard the discovery of Dr. Hullihen as among the most valuable improvements in dental surgery.

